# Analysis of the efficacy of laparoscopic high hernia sac ligation in adolescent indirect hernia

**DOI:** 10.1186/s12893-023-02048-w

**Published:** 2023-06-13

**Authors:** Guoyong Fan, Gan Yao

**Affiliations:** Department of Hernia and Abdominal Wall Surgery, The First People’s Hospital of Foshan, Address: No. 81, Lingnan Avenue North, Foshan, 528000 China

**Keywords:** Indirect hernia, Adolescent, Laparoscopic, High hernia sac ligation

## Abstract

**Objective:**

Inguinal hernias are a common disease that may present at all ages. Adolescents are a unique patient population between children and adults. The etiology and the surgical treatment strategies of adolescent indirect hernias are not clear. Specifically, whether these kinds of hernias should be treated by high ligation or mesh repair remains controversial. We aimed to evaluate the efficacy of laparoscopic high hernia sac ligation in adolescent indirect hernia.

**Methods:**

The data of adolescent patients who underwent laparoscopic high hernia sac ligation at the The First People’s Hospital of Foshan,China, from January 2012 to December 2019 were analyzed retrospectively. Data collected included their age, gender, weight, surgical method, hernia ring diameter, operation time, postoperative recurrence rate and postoperative complications.

**Results:**

A total of 70 patients were enrolled, including 61 males (87.14%) and 9 females (12.86%),all patients were aged 13–18 years old (mean 14.87 ± 1.56years), weighed 28-92 kg (mean 53.04 ± 10.60 kg). All 70 patients underwent laparoscopic surgery except 2 patients with irreducible hernias who converted to laparotomy.The hernia ring diameter ranged from 0.5 to 3 cm (mean 1.39 ± 0.49 cm), and was ≤ 2 cm in 68 cases (97.14%).The operative length ranged from 12 to 105 min (average 24.96 ± 12.61 min), There were 37(52.9%) right-sided hernias, 32 (45.7%) left-sided hernias, 1 (1.4%) bilateral hernia.There were 67(95.7%)reducible hernias,2(2.9%)irreducible hernias ,and 1(1.4%)incarcerated hernia.Hospital stays ranged from 1 to 5 days (mean 2.37 ± 1.05 days). Follow-ups were performed from 30-119months (mean 74.27 ± 28.14months). There were no cases of recurrence, howere, incision infection occured in 1 patient, who underwent a second operation 6 months after surgery, and 4 (5.7%) patients had complaints of intermittent pain around the ligation incision site, mostly during exercise .

**Conclusion:**

Laparoscopic high hernia sac ligation is feasible for treatment of adolescent indirect hernias with a hernia ring diameter of ≤ 2 cm.

**Supplementary Information:**

The online version contains supplementary material available at 10.1186/s12893-023-02048-w.

Inguinal hernias are a commom disease that may present at all ages, The overall incidence of inguinal hernias in childhood ranges from 0.8–4.4% [1].Indirect hernias in children are a result of incomplete closure of the processus vaginalis [2]. The surgical treatment for an indirect inguinal hernia in children is high ligation. In addition the incidence of adult inguinal herniorrhaphy is 27-42.5% in males and 3-5.8% in females [3–6]. Adult inguinal hernias are a result of defects in the transversalis fascia due to weakness [7]. Therefore, the standard treatment for adult inguinal hernias are posterior wall repair and tension-free repair with mesh, which is most commonly used treatment. Of note adolescents are a unique patient population between children and adults. The etiology and the surgical treatment strategies of adolescent indirect hernias are not clear. Specifically,whether these kinds of hernias should be treat by high ligation or mesh repair remains controversial.This study retrospectively analyzed the clinical data of laparoscopic high hernia sac ligation in the treatment of adolescent indirect hernia. The long-term outcomes were evaluated to validate the efficacy of laparoscopic high hernia sac ligation for adolescent indirect hernias .

## Materials and methods

We retrospectively reviewed the data of adolescent patients who underwent inguinal hernia repairs from January 2012 to December 2019 at The First People’s Hospital of Foshan, Guangdong, China. The inclusion criteria for this study were: age 13–18 years;primary indirect inguinal hernia; signed informed consent by the patient’s legal guardian; and no dysfunction of vital organs, including the heart, lungs, liver, and kidneys. Exclusion criteria were: age < 13 years or > 18 years; direct hernia; femoral hernia; recurrent hernia; patients who refused to sign informed consent; and heart, lung, liver, and kidney failure. The gender, age ,weight, hernia characteristics, operative approach, operative length,length of stay, postoperative recurrence rate, and complications of the patients were collected.Postoperative complications were defined as surgical site infection, symptomatic seroma or hematoma, symptomatic hydrocele, postoperative pain.

### Surgical method

**Fig. 1:**
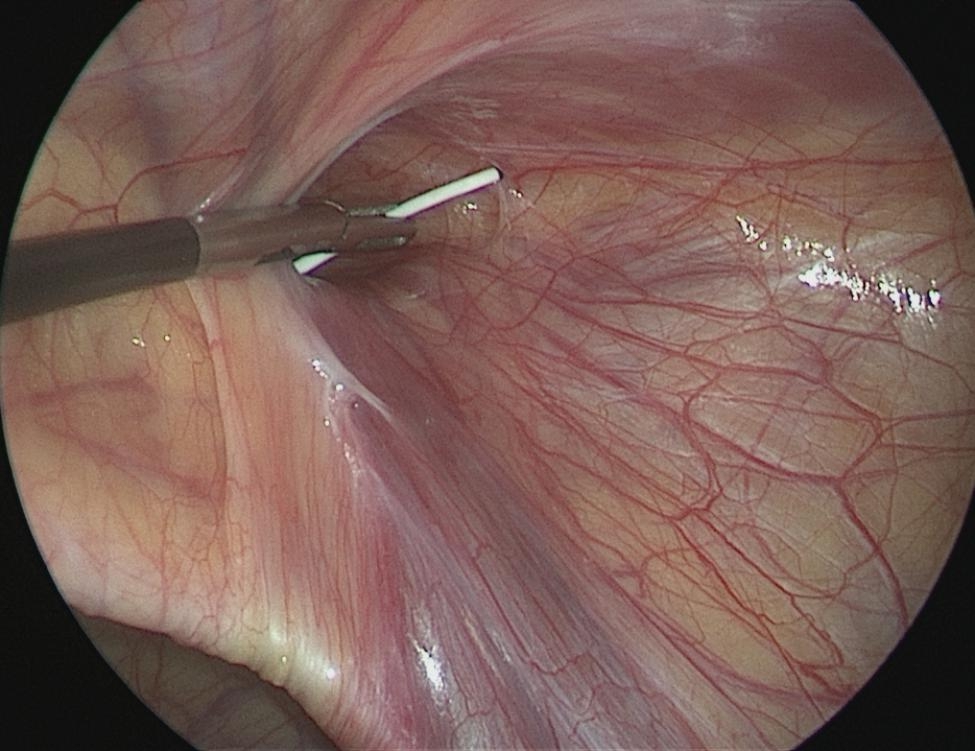
The size of the hernia diameter was measured

**Fig. 2:**
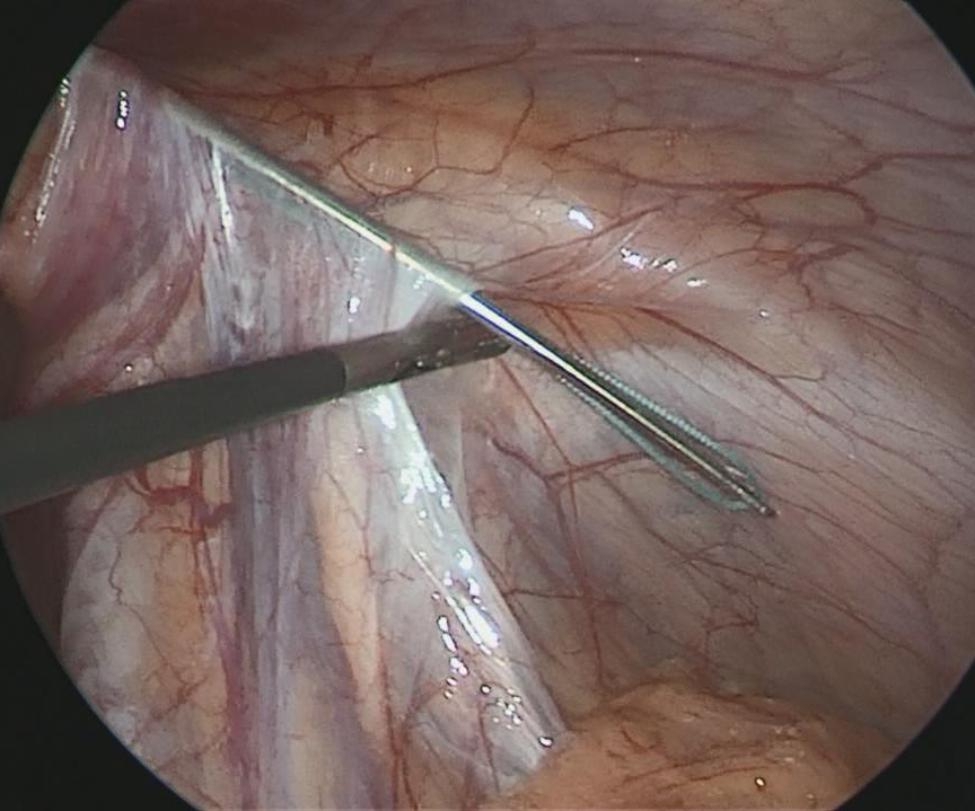
The sled needle with a 2 − 0 non-absorbable suture was puncture into the abdominal cavity

**Fig. 3:**
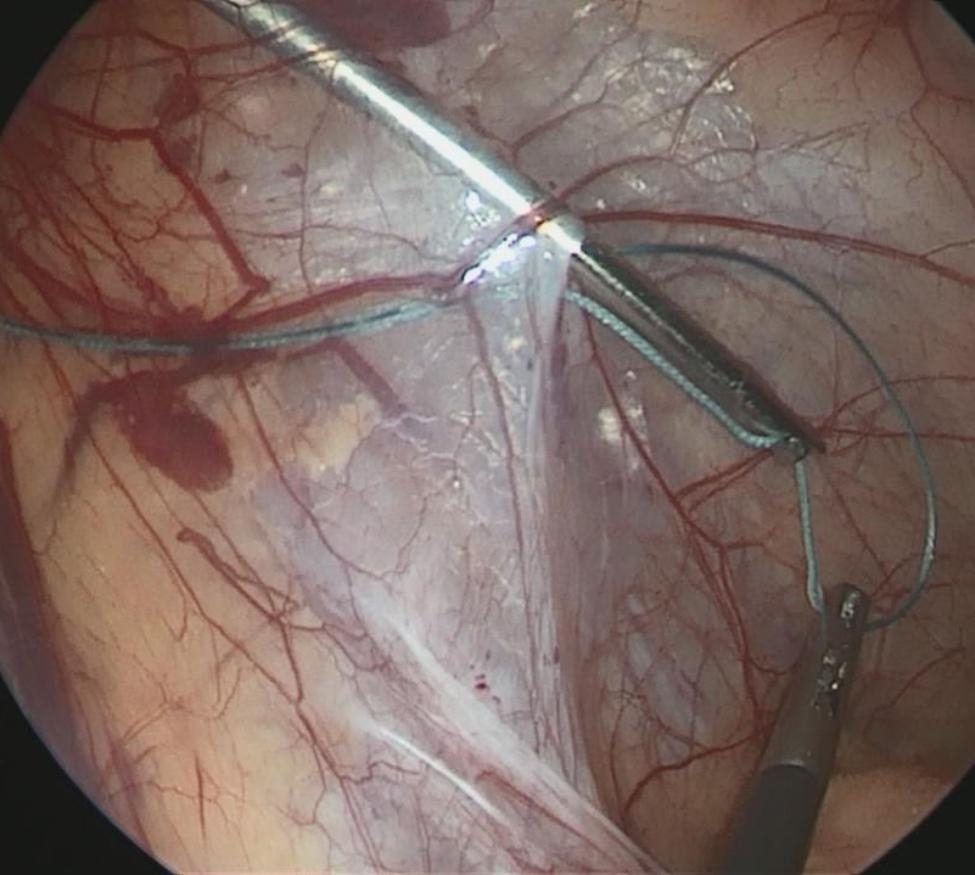
The suture was hooked and took out of the body surface by the crochet

**Fig. 4:**
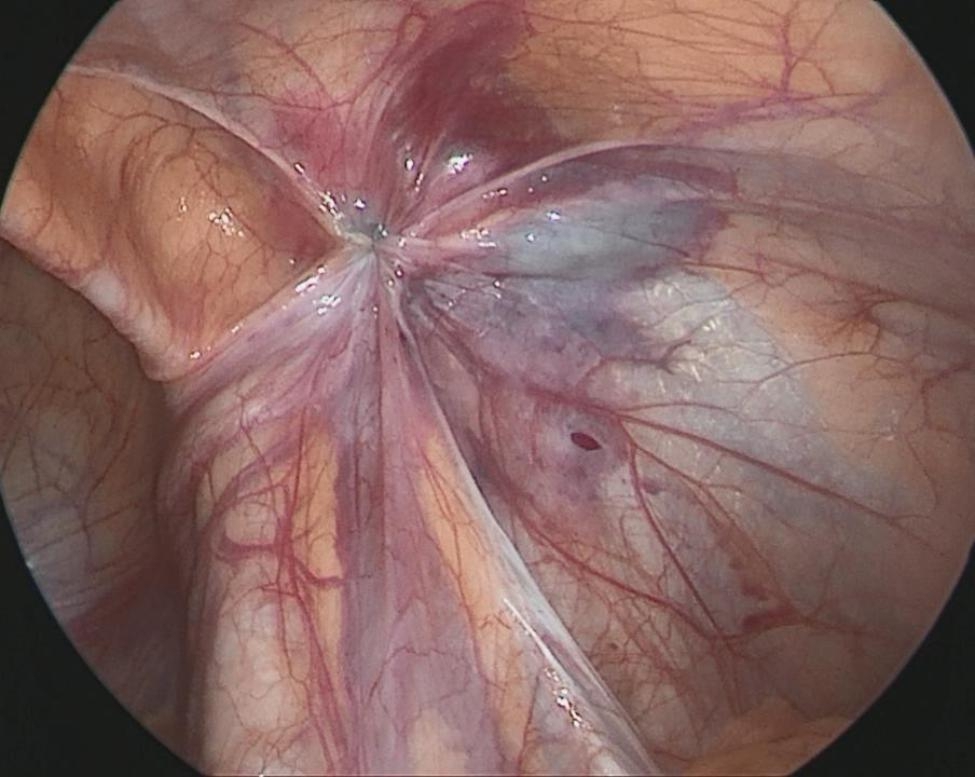
The high ligation of the hernia sac was complete

The procedures of laparoscopic high hernia sac ligation were performed with the patient in the supine position under general anesthesia. The typical process of the procedure is described as follows:

The size of the hernia diameter was measured (Fig. 1). A 2 mm incision was made on the surface body of the deep ring, and a sled needle with a 2 − 0 non-absorbable suture was punctured into the preperitoneal space, which is through the medial peritoneal membrane of the deep ring and the vascular surface, to the bottom of the deep ring. The needle was then punctured into the abdominal cavity (Fig. 2). The suture was left in the abdominal cavity, and the needle was withdrawn. A crochet was inserted through the original incision, through the lateral peritoneal membrane of the deep ring, and punctured into the abdomen through the original puncture hole. The crochet hooked the suture, which was then taken out of the bodies surface (Fig. 3). The suture was cut into two and double-knotted subcutaneously; the high ligation of the hernia sac was completed (Fig. 4).

### Follow-ups

Patients were followed up by the outpatient department at 1 week, 1 month and 3 months post-operation. At 3 months post-operation, patients were followed up by telephone. Patients were asked yes and no questions whether they had any residual pain at the surgical incision site, bulging in the groin, or whether they had surgical site redness and any discharge of pus. If recurrence was suspected, the patient would be called back to the outpatient department and excluded by physical examination or imaging .All patients were followed-up. The follow-up time of the entire cohort was 30-119months (mean 74.27 ± 28.14months).

### Statistical method

All statistical analyses were performed using the spss21 statistical software .Continuous data were expressed as the mean ± standard deviation.

## Results

A total of 70 patients were enrolled, including 61 males (87.14%) and 9 females (12.86%); all patients were aged 13–18 years (mean14.87 ± 1.56years), and their body weight was between 28 and 92 kg(mean 53.04 ± 10.60 kg). 68 patients underwent elective laparoscopic high hernia sac ligation except 1 incarcerated hernia underwent emergency laparoscopic surgery. In addition, 2 patients underwent laparoscopic surgery converted to laparotomy because of irreducible hernias.The hernia ring diameters for the patients were between 0.5 and 3 cm (mean 1.39 ± 0.49 cm); among these hernia rings of 1 patient was 3 cm,another patient was 2.5 cm, while 68(97.14%) patients had hernia rings ≤ 2 cm .The operative lengths were between 12 and 105 min (average 24.96 ± 12.61 min). There were 37(52.9%) right-sided hernias, 32 (45.7%) leftsided hernias, and 1 (1.4%) bilateral hernia. Moreover, 24 patients(34.3%) simultaneously had contralateral hernias.There were 67 (95.7%)reducible hernias, 2 (2.9%)irreducible hernias, and 1 (1.4%)incarcerated hernia (Table 1). Hospital stay was between1-5 days for all patients (mean 2.37 ± 1.05 days). Follow-ups 30-119months (mean 74.27 ± 28.14months) with no hernia recurrence, however, incision infection did occur in one patient, who underwent a second operation 6 months post-surgery, and 4 (5.7%) patients had complaints of intermittent pain around the ligation incision site, particularly during exercise. There were no documented occurrances of hydroceles ,hematomas or seromas.

**Table 1:** Patient characteristics

	High ligation(*N* = 70)
Age(years)	14.87 ± 1.56
Sex	
Male	61(87.14%)
Female	9 (12.86%)
Body weight(kg)	53.04 ± 10.60(28–92)
Laterality	
Right	37(52.9%)
Left	32 (45.7%)
Bilaterally	1 (1.4%)
Hernia content	
reducible	67 (95.7%)
irreducible	2(2.9%)
incarcerated	1(1.4%)
Defect size (cm)	1.39 ± 0.49 (0.5-3 ),
Follow up(months)	74.27 ± 28.14 (30–119)

## Discussion

At present, there is no consensus on the cause and surgical treatment of adolescent indirect hernias. One opinion is that adolescent indirect hernias are considered to be the continuation of indirect hernias present in children. Some of these cases are perhaps those who develop symptoms early in childhood but are not treated until adolescence, and some may be from those born with incomplete closure of the processus vaginalis who do not show clinical symptoms until adolescence. The treatment for an adolescent indirect inguinal hernia is high ligation. Another main opinion is that post-pubescent adolescents have similar anatomy to that of adults, with a larger internal ring diameter and variations on transverse fascia defects [7]. Therefore, the indirect hernias of adolescents need to be repaired with tissue or mesh to strengthen the posterior inguinal wall.

Whether high ligation or mesh repair is more appropriate for the treatment of adolescent hernias mainly depends on the postoperative recurrence rates. Most recurrences are likely to occur within 2 years of inguinal hernia repair, but the recurrence rates may increase with the extension of follow-up times [8–13].The recurrence rate of inguinal hernia after operation is 0-6.3% [14–16]. Tension-free mesh repair is helpful in reducing recurrence rates and has become the mainstream operation for adult hernias. However, some investigators raised the concern of an increased risk of foreign body rejection and infection after placement of a mesh in adolescents, which may affect the function of vas deferens [17, 18]. Therefore, If the recurrence rates do not increase with high ligation alone, especially for those patients with a small internal ring, a mesh may be avoided for adolescent hernia patients. Although one study on the treatment of adolescent hernias emphasized the likelihood that the surgical procedure was driven by the surgeon’s preferences and not necessarily the course of the disease or the patient outcome [19], the reason for this may be due to the rarity of studies on the treatments of adolescent hernias.

In this study, a retrospective analysis was carried out to evaluate the efficacy of laparoscopic high hernia sac ligation in the treatment of adolescent indirect hernias. After 30-119months (mean 74.27 ± 28.14months) of follow-ups, the recurrence rate were 0%. The observed positive outcome of the present series may be due to the following reasons: (1) patient selection, we included patients with small internal rings (1.39 ± 0.49cm); (2) all patients received double high hernia sac ligation without peritoneal tear. Some other studies have shown that the recurrence rates after high hernia sac ligation without mesh for adolescent hernias were 0.95-3.5%, which is also relatively low [7, 20].The recurrence rates for adolescent hernias after high ligation were similar to those after mesh repair for adult hernias [21]. Another study reported that the recurrence rates in patients aged 18–25 years who underwent herniectomy without mesh placement or posterior wall repair were 0% [22]. These results suggest that high hernia sac ligation without mesh placement or posterior wall repair is effective in treating adolescent hernias.However, none of these studies showed the size of the deep ring of the indirect hernia, as the size of the internal ring may affect the surgeon’s choice of which treatment approach to adhere to [17, 23]. If the internal ring is small, the surgeon may prefer the hernia sac high ligation, but if the internal ring is large, the surgeon may prefer the mesh repair or tissue repair approach over others.The cut-off point of the internal ring size must be determined to guide surgeons in selecting the appropriate surgical procedure.S. R. Lee. et al [20] did a comparative study of high ligation of the laparoscopic hernia sacs and posterior wall repairs in adolescent indirect hernias. The size of the internal ring diameter in the two groups was 2.37 ± 0.52 (1.60–3.62) cm versus 2.41 ± 0.58 (1.59–3.65) cm (p = 0.592). The results showed that the recurrence rates in the high ligation group(4/115,3.48%) were higher than that in the posterior wall suture repair group (0/129, 0%) (p = 0.048). Shen Yingmo et al. [24] carried out a prospective comparative study between high ligation and Lichtenstein hernioplasty using acellular tissue matrix grafts in adolescent patients. The hernia ring diameters of the two groups were 2.2 ± 0.5 cm and 2.3 ± 0.5 cm(p = 0.499), respectively The results showed that there were no difference in the recurrence rates between the two groups6% (3/50) versus 0% (0/50) (p = 0.079) respectively, but the subgroup (the patients with Gilbert type 3 hernias)analysis found that the recurrence rates in the high ligation group were higher than those in the mesh repair group. While using a biologic mesh for a Lichtenstein repair is not the care standard, the results indicated that mesh repair may be a more suitable surgical method in adolescent with an indirect hernia, an internal hernia ring diameter greater than 3 cm, and severe transverse fascia defect.Based on the current literature [20, 24], mesh repair treatment is recommended when the diameter of the internal hernia ring is > 3 cm or there is severe transverse fascia defect in adolescent indirect hernias. Evidently, when the diameters of the internal hernia ring between 2 and 3 cm, the efficacy of high hernia sac ligations was controversial, and these patients could be considered for laparoscopic posterior wall suture repair or open muscle repair. In our study, 97% of adolescent indirect hernia patients with an internal ring diameter ≤ 2 cm were treated with laparoscopic high hernia sac ligation, and the recurrence was zero. Therefore, we believe that high hernia sac ligation in adolescents with an internal hernia ring diameter of ≤ 2 cm is effective.

The main complication after inguinal hernia repair is seroma, but since the high ligation of the hernia sac did not require the separation of the preperitoneal space and hernia sac, the incidence of seroma was low. No seroma occurred in the paients analyzed in this study. One case of incision infection was documented. The patient had undergone a laparoscopy converted to laparotomy because of an irreducible hernia. However, 6 months post-operation, the wound became infected due to the non-absorbable suture knots which were removed by a second operation. In particular, 4 patients (4/70,5.7%) had complaints of intermittent pain around the ligation incision site, mostly during exercise. This was similar to previous reports [7, 25]. The patients’ pain degrees were mild, so no treatments were needed. Finally, no hydrocele occurred after surgery.

There were some limitations in our study; the small number of cases, no comparative study was conducted, and the follow-up times of some patients were short compared to others. Follow-ups of patients who recently underwent surgery should be conducted over a longer period of time to observe the true recurrence rates. Prospective muti-center studies with larger sample size should be designed in the future.

## Conclusion

The results of this study suggest that laparoscopic high hernia sac ligations are effective in the treatment of adolescent indirect hernias with internal ring diameters of ≤ 2 cm. Moreover, the rates of recurrence and complications are low, and there is no need to worry about the adverse effects of using mesh. However, this study is a retrospective study with a small number of participants. Further prospective comparative studies are required.

## Electronic supplementary material

Below is the link to the electronic supplementary material.

Additional File 1: The data of adolescent hernias

## Data Availability

The datasets used and/or analysed during the current study available from the corresponding author on reasonable request.
