# Supplementary material for: Analysis of the efficacy of laparoscopic high hernia sac ligation in adolescent indirect hernia
Source: BMC Surg. 2023 Jun 13;23:160. doi: 10.1186/s12893-023-02048-w (PMC10265881; doi:10.1186/s12893-023-02048-w)
Supplement: Supplementary file 1 — Additional File 1: The data of adolescent hernias [file 12893_2023_2048_MOESM1_ESM.docx]

The data of adolescent hernias

| Case | Age(y) | Gender | Weight(kg) | Operative approach | Affected side | The hernia ring diameter(cm) | Contralateral hernias | The operative length(min) | Hospital stay(d) | Follow up(months) | Infection | Postoperative pain |
| --- | --- | --- | --- | --- | --- | --- | --- | --- | --- | --- | --- | --- |
| 1 | 14 | male | 46 | LHSL | left | 1 |  | 15 | 2 | 30 |  |  |
| 2 | 14 | male | 56 | LHSL | left | 1 |  | 20 | 3 | 30 |  |  |
| 3 | 18 | male | 68 | LHSL | left | 0.5 | right | 30 | 2 | 32 |  |  |
| 4 | 14 | female | 61 | LHSL | left | 0.5 |  | 25 | 1 | 33 |  |  |
| 5 | 17 | male | 59 | LHSL | right | 2 | left | 30 | 2 | 35 |  |  |
| 6 | 13 | male | 36 | LHSL | left | 1 |  | 35 | 2 | 35 |  |  |
| 7 | 13 | female | 43 | LHSL | right | 0.5 | left | 20 | 1 | 39 |  |  |
| 8 | 15 | male | 51 | LHSL | right | 2 |  | 27 | 2 | 39 |  |  |
| 9 | 15 | male | 55.5 | LHSL | right | 1.8 |  | 15 | 1 | 40 |  |  |
| 10 | 16 | male | 50 | LHSL | bilaterally | 1；1* |  | 30 | 2 | 40 |  |  |
| 11 | 15 | female | 36 | LHSL | right | 1 | left | 35 | 3 | 40 |  |  |
| 12 | 17 | male | 56 | LHSL | left | 1 |  | 20 | 2 | 41 |  |  |
| 13 | 14 | male | 47 | LHSL | left | 2 | right | 25 | 2 | 41 |  |  |
| 14 | 13 | male | 60 | Laparoscopic surgery converted to open | right | 1.5 |  | 105 | 1 | 41 |  |  |
| 15 | 14 | male | 44 | LHSL | left | 1 | right | 35 | 2 | 46 |  |  |
| 16 | 16 | male | 52 | LHSL | left | 1 |  | 50 | 3 | 47 |  |  |
| 17 | 14 | male | 41 | LHSL | left | 1 |  | 15 | 3 | 52 |  |  |
| 18 | 13 | male | 47 | LHSL | left | 1 |  | 22 | 1 | 52 |  |  |
| 19 | 15 | male | 92 | LHSL | right | 2 |  | 30 | 2 | 52 |  | pain |
| 20 | 16 | male | 56.5 | LHSL | right | 1.5 |  | 15 | 2 | 53 |  | pain |
| 21 | 17 | male | 56 | LHSL | right | 1.8 |  | 20 | 2 | 53 |  |  |
| 22 | 16 | male | 56 | LHSL | left | 1 | right | 25 | 3 | 53 |  |  |
| 23 | 18 | male | 58 | LHSL | left | 1 |  | 30 | 3 | 53 |  |  |
| 24 | 13 | male | 45 | LHSL | left | 1 |  | 45 | 3 | 53 |  |  |
| 25 | 14 | male | 50 | LHSL | right | 1.5 |  | 25 | 2 | 57 |  |  |
| 26 | 14 | male | 49 | LHSL | left | 1.5 |  | 45 | 1 | 57 |  |  |
| 27 | 13 | male | 39 | LHSL | right | 2 | left | 25 | 2 | 58 |  |  |
| 28 | 13 | male | 45 | LHSL | left | 1 |  | 30 | 3 | 58 |  |  |
| 29 | 14 | male | 50.5 | LHSL | left | 1 |  | 25 | 2 | 59 |  |  |
| 30 | 13 | male | 44.5 | LHSL | left | 1.5 | right | 15 | 2 | 61 |  |  |
| 31 | 17 | male | 47.5 | LHSL | right | 1.8 |  | 20 | 3 | 65 |  |  |
| 32 | 13 | female | 62 | LHSL | right | 1.5 |  | 40 | 2 | 65 |  | pain |
| 33 | 13 | male | 52.5 | LHSL | right | 1.5 |  | 12 | 1 | 70 |  |  |
| 34 | 16 | male | 88 | LHSL | right | 2.5 |  | 15 | 3 | 71 |  |  |
| 35 | 14 | male | 58 | LHSL | right | 2 | left | 25 | 1 | 71 |  |  |
| 36 | 15 | female | 47.5 | LHSL | left | 1.5 |  | 20 | 1 | 77 |  |  |
| 37 | 16 | male | 49 | LHSL | right | 1 |  | 15 | 1 | 78 |  |  |
| 38 | 14 | male | 49 | LHSL | right | 1.5 | left | 30 | 1 | 80 |  |  |
| 39 | 15 | female | 54.5 | LHSL | left | 1 | right | 23 | 1 | 82 |  |  |
| 40 | 17 | male | 46 | LHSL | right | 2 | left | 29 | 1 | 84 |  |  |
| 41 | 18 | male | 58 | LHSL | right | 2 |  | 20 | 2 | 87 |  |  |
| 42 | 13 | male | 47 | LHSL | right | 2 |  | 15 | 1 | 88 |  |  |
| 43 | 18 | male | 57 | LHSL | right | 1 |  | 15 | 3 | 88 |  |  |
| 44 | 14 | male | 58 | LHSL | left | 1 |  | 15 | 2 | 88 |  |  |
| 45 | 14 | male | 65.5 | LHSL | right | 2 |  | 20 | 4 | 88 |  |  |
| 46 | 16 | male | 56 | LHSL | right | 1.5 |  | 15 | 2 | 89 |  |  |
| 47 | 16 | male | 50 | LHSL | right | 2 | left | 25 | 1 | 89 |  |  |
| 48 | 14 | male | 45 | LHSL | left | 1 | right | 23 | 3 | 90 |  |  |
| 49 | 14 | male | 68 | LHSL | right | 1.5 |  | 15 | 4 | 93 |  |  |
| 50 | 16 | male | 60 | LHSL | left | 1.5 |  | 18 | 3 | 95 |  |  |
| 51 | 15 | male | 56 | LHSL | left | 1.5 | right | 25 | 3 | 96 |  |  |
| 52 | 15 | female | 41 | LHSL | left | 1 |  | 15 | 3 | 100 |  |  |
| 53 | 14 | female | 44 | LHSL | left | 1 | right | 32 | 4 | 100 |  |  |
| 54 | 13 | male | 56 | LHSL | right | 1.5 | left | 25 | 1 | 101 |  |  |
| 55 | 13 | male | 43 | LHSL | right | 1.5 | left | 28 | 3 | 101 |  |  |
| 56 | 17 | female | 42.5 | LHSL | right | 1.5 | left | 30 | 2 | 101 |  |  |
| 57 | 16 | male | 60 | LHSL | right | 1 |  | 20 | 3 | 106 |  |  |
| 58 | 13 | male | 49 | Laparoscopic surgery converted to open | left | 1 |  | 25 | 1 | 107 | incision infection | pain |
| 59 | 16 | male | 49.2 | LHSL | left | 2 |  | 25 | 2 | 108 |  |  |
| 60 | 14 | male | 53.5 | LHSL | left | 1 |  | 20 | 4 | 112 |  |  |
| 61 | 14 | male | 62.5 | LHSL | left | 1 |  | 20 | 4 | 112 |  |  |
| 62 | 13 | male | 28 | LHSL | left | 1 | right | 30 | 4 | 112 |  |  |
| 63 | 15 | male | 55 | LHSL | right | 1 | left | 30 | 5 | 112 |  |  |
| 64 | 14 | male | 38 | LHSL | right | 1.5 |  | 15 | 4 | 113 |  |  |
| 65 | 16 | male | 77 | LHSL | right | 2 |  | 15 | 4 | 113 |  |  |
| 66 | 18 | male | 54.5 | LHSL | right | 1 |  | 25 | 3 | 113 |  |  |
| 67 | 15 | male | 63 | LHSL | left | 3 |  | 15 | 3 | 118 |  |  |
| 68 | 16 | male | 60 | LHSL | right incarcerated** | 2 | left | 21 | 3 | 118 |  |  |
| 69 | 17 | male | 58 | LHSL | right | 1 |  | 12 | 4 | 119 |  |  |
| 70 | 13 | male | 54 | LHSL | right | 1.5 | left | 25 | 4 | 119 |  |  |

LHSL: Laparoscopic high hernia sac ligation.

*: The patient (case 10) presented with bilateral hernias, both of which had a hernia ring diameter of 1cm.

**: The patient (case68) presented with a right incarcerated hernia.
